# Harnessing Magnetic Properties for Precision Thermal Control of Vortex Domain Walls in Constricted Nanowires

**DOI:** 10.3390/nano15050372

**Published:** 2025-02-27

**Authors:** Mohammed Al Bahri, Salim Al-Kamiyani

**Affiliations:** Department of Basic and Applied Sciences, A’Sharqiyah University, P.O. Box 42, Ibra 400, Oman

**Keywords:** micromagnetic simulation, vortex domain wall, confined magnetic nanowire, spin transfer torque, VW thermal stability

## Abstract

This study investigates the thermal pinning and depinning behaviors of vortex domain walls (VWs) in constricted magnetic nanowires, focusing on the influence of intrinsic magnetic properties on VW stability under thermal stress. Using micromagnetic simulations, we analyze the roles of saturation magnetization (Ms), uniaxial magnetic anisotropy (Ku), and nanowire geometry in determining VW thermal stability. The modeled nanowire has dimensions of 200 nm (width), 30 nm (thickness), and a 50 nm constriction length, chosen based on the dependence of VW formation on nanowire geometry. Our results show that increasing *Ms* and *K_u_* enhances VW pinning, while thermal fluctuations at higher temperatures promote VW depinning. We demonstrate that temperature and magnetic parameters significantly impact VW structural stability, offering insights for designing high-reliability nanowire-based memory devices. These findings contribute to optimizing nanowire designs for thermally stable, energy-efficient spintronic memory systems.

## 1. Introduction

The swift advancement of spintronic technologies has established magnetic nanowires as promising candidates for energy-efficient, low-power data storage. Their small size and unique magnetic properties make them ideal for high-density and energy-efficient memory devices. Vortex domain walls (VWs), with flux-closure structures and minimized stray fields, enhance memory stability and reduce energy consumption. These properties make VWs particularly suitable for next-generation spintronic devices, especially high-density memory systems. Efficient VW manipulation in confined geometries is crucial for increasing storage capacity [1,2,3].

VW-based memory systems depend on the structural stability of VWs, which affects retention, reliability, and energy efficiency [4,5,6,7]. A stable VW ensures consistent pinning and controlled depinning, preventing unwanted transformations that cause data loss or higher power consumption. Temperature variations, saturation magnetization (*M_s_*), uniaxial anisotropy (*K_u_*), and nanowire geometry play key roles in maintaining VW stability. Optimizing these factors enhances spintronic memory performance and longevity [8,9,10,11].

VW motion is governed by pinning and depinning, determining whether a VW remains fixed or moves under external forces such as applied current or magnetic fields. Pinning stabilizes VWs, while depinning enables controlled propagation for data transfer [12,13,14]. At high temperatures, VWs can become unstable and transform into transverse domain walls (TWs) due to Walker breakdown, which occurs when velocity surpasses a critical threshold, leading to oscillatory motion and unpredictable transformations [15,16]. This instability poses a challenge in nanoscale devices, where precise VW control is essential for data integrity [17,18,19].

Previous studies addressed these limitations through different approaches. Theoretical simulations at 0 K eliminate temperature effects but fail to capture thermal-induced depinning, deformations, and transitions at elevated temperatures [20,21]. Experimental studies using microscale devices or thin films to minimize temperature effects do not accurately reflect nanoscale domain wall interactions and pinning dynamics [22,23,24,25].

To address these challenges, we systematically investigate VW thermal stability in constricted nanowires with stepped areas using micromagnetic simulations. Our study focuses on nanowire-based architecture to ensure practical relevance for nanoscale memory and spintronic systems. A key advancement is the introduction of a stepped nanowire structure for pinning. Unlike traditional notches, which introduce edge defects that destabilize VWs, the stepped design provides effective pinning while preserving VW structure, enhancing thermal stability and information retention. The absence of a central edge reduces unwanted depinning events, prolonging VW lifespan in memory applications.

The stepped nanowire allows for the precise control of pinning strength by adjusting step depth (*d*) and length (λ), optimizing storage density and energy efficiency. This scalable design also enables multiple stepped or constricted regions, supporting multi-bit storage and increasing memory capacity.

By addressing thermal, geometric, and dynamic challenges, our work enhances VW stability, pinning efficiency, and memory device performance. Prior studies focused on modifying nanowire dimensions, which influence VW behavior but struggle to control temperature effects and ensure long-term stability under thermal fluctuations.

Our study takes a material-based approach, investigating how *M_s_* and Ku improve VW stability and pinning against temperature variations. Increasing *M_s_* or Ku enhances VW thermal stability and pinning behavior. Optimizing step depth (*d*) and material properties provides an effective strategy for controlling domain wall motion and mitigating Walker breakdown due to thermal fluctuations.

This study integrates foundational research with recent advancements, focusing on three key factors: (1) the influence of *M_s_* and *K_u_* on VW thermal stability, (2) the effect of temperature on VW behavior under optimized Ku and *M_s_*, and (3) the interplay of *K_u_*, *M_s_*, and stepped nanowire geometry (*d*) in VW thermal pinning and depinning. These findings contribute to the development of stable, energy-efficient VW-based memory systems.

Magnetic nanowires, commonly made from Fe, Co, Ni, and their alloys, exhibit high spin polarization and strong anisotropy, making them ideal for domain wall-based memory and logic applications. Using micromagnetic simulations, we investigate VW thermal pinning and depinning, which are critical for reliable spintronic memory devices. Mesoscopic ab initio modeling [26] offers atomic-scale insights into nanowire properties, refining the predictions of thermal stability and domain wall behavior. Future multiscale simulations integrating micromagnetic and ab initio approaches [27] could further optimize nanowire design, bridging theoretical and experimental advancements.

## 2. Theoretical Model

Magnetic simulations were carried out using the Object Oriented Micro-Magnetic Framework (OOMMF) [28], which solves the Landau–Lifshitz–Gilbert (LLG) equation [29]. When the current flows along the wire axis, the LLG equation can be written as follows:

(1) $$\frac{dm}{dt}=-\gamma m\times H_{eff}+H_{th}+\alpha m\times \frac{dm}{dt}-\left(u\cdot \nabla \right)m+\beta m\times \left(u\cdot \nabla \right)$$

where m is the unit vector of the magnetization, γ is the gyromagnetic ratio, $H_{eff}$ is the effective magnetic field, α is the Gilbert damping constant, and ζ(T) is the thermal field, which depends on the device temperature (*T*),

$$u=\frac{JPg\mu_{B}}{2eM_{s}}$$

where *J*, *P*, *g*, *μ_B_*, and *M*_s_ are the current density, spin polarization rate, g-factor of the electron, Bohr magneton, electron charge, and saturation magnetization, respectively [4,29]. The thermal field varies with device temperature according to the following:

(2) $$\langle H_{th,i}\left(r,t\right),H_{th,j}(\overset{´}{r},\overset{´}{t})\rangle =\frac{2\alpha k_{B}T}{\gamma \mu_{0}M_{s}V}\delta_{ij}\delta (r-\overset{´}{r})\delta (t-\overset{´}{t})$$

where $k_{B}$ is the Boltzmann constant, $\mu_{0}$ is the vacuum permeability, and V is the cell volume [29].

In this research, the dimensions of the confined nanowire are characterized by its length (*l*), width (*w*), and thickness (*th*), specified as 1000 nm × 200 nm × 30 nm. The confined region is characterized by its height (*d*) and length (*λ*), defined as *d* nm × 50 nm. This study utilized the magnetic properties of a material with in-plane magnetization [30]. The uniaxial magnetic anisotropy (*K_u_*) is directed along the longitudinal axis (*x*-axes) of the nanostrip (parallel to the 1000 nm length). This configuration aligns the magnetization with the nanowire’s geometry, facilitating stable vortex domain wall propagation. For precision, a cubic mesh with a unit cell dimension less than the exchange length (*l*_ex_ = 5.3 nm) was necessary, and a unit cell size of 5 nm was consistently applied across all simulations [31].

## 3. Results and Discussion

Domain wall pinning increases with higher saturation magnetization (*M_s_*) and uniaxial magnetic anisotropy constant (*K_u_*) due to their direct effects on the energy and forces governing domain wall dynamics. The pinning energy at a pinning site can be expressed as follows:

*E*_pin_ = C*µ*_0_*M*_*s*_^2^*V* (3)

where C is a proportionality constant that depends on the specific material and geometric factors of the system, *µ*_0_ is the permeability of free space, and *V* is the volume of the pinning site [32].

This quadratic dependence on *M_s_* indicates that as *M_s_* increases, the magnetic stiffness and the energy barrier for domain wall motion rise, making depinning more challenging. Similarly, the pinning energy scales linearly with *K_u_* as follows:

*E*_pin_ = C*K*_*u*_*V* (4)

where C is a proportionality constant that depends on the specific material and geometric factors of the system, *K_u_* is a uniaxial magnetic anisotropy constant, and *V* is the volume of the pinning site [33].

These relationships underscore that both *M_s_* and *K_u_* significantly enhance domain wall pinning by increasing energy barriers and stabilizing the domain wall at pinning sites, as observed in micromagnetic simulations and experimental studies [34,35].

**Figure 1:**
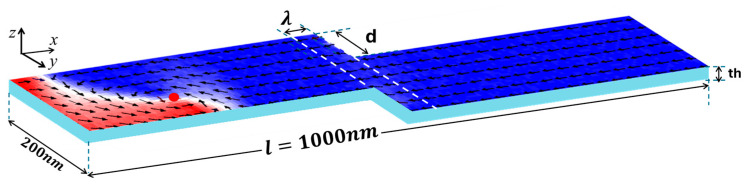
The confined nanowire’s dimensions for VW thermal stability analysis include a length of 1000 nm, a width of 200 nm, and a thickness of 30 nm. The VW is nucleated at a critical current density and propagates toward the confined region.

In this study, the thermal stability of vortex domain walls (VWs) was analyzed based on magnetic properties such as *M_s_* and *K_u_*. We modeled an advanced 3D storage memory system that leverages VW pinning for efficient data writing and storage within a single confined nanowire. As VW pinning is influenced by device temperature, simulations were conducted at various temperatures to assess the thermal stability of the VW structure during motion and within the confined region, emphasizing the pinning and depinning processes. A constant current density was applied to facilitate VW movement along the confined nanowire. Figure 1 illustrates the dimensions of the confined nanowire, which were used to examine the thermal effects on the VW. The VW is generated at a critical current density and propagates toward the confined area.

This study examines the behavior of vortex domain walls (VWs) under different thermal conditions, with a focus on three key aspects: (1) how VWs undergo structural changes due to thermal effects during motion, (2) the influence of device temperature on VW movement and dynamics, and (3) the processes of thermal pinning and depinning of VWs within the confined region.

### 3.1. VW Thermal Structural Stability

**Figure 2:**
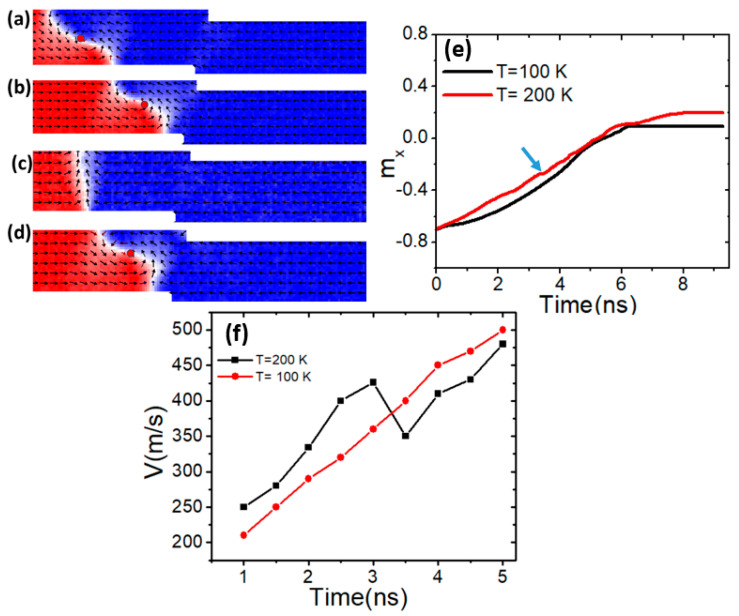
(**a**,**b**) VW dynamics in constricted nanowire with *M_s_* = 600 kAm^−1^, a device temperature of 100 K, and a current density of 7.5 × 10^11^ Am^−2^. (**c**) VW transformation to TW under *M_s_* = 600 kAm^−1^, a device temperature of 200 K, and a current density of 7.5 × 10^11^ Am^−2^. (**d**) VW motion with *M_s_* = 650 kAm^−1^ and under a device temperature of 200 K. (**e**) *m_x_* versus time for two curves with different device temperature values of 100 K and 200 K. (**f**) DW velocity at two device temperatures.

One of the magnetic properties that affects the thermal transformation of vortex domain walls (VWs) is saturation magnetization (*M_s_*). In this study, we investigate how controlling VW thermal transformation can be achieved by adjusting *M_s_*. Figure 2a shows the dynamics of VWs in magnetic nanowires with an *M_s_* of 600 kAm^−1^ under a device temperature of 100 K and a current density of 7.5 × 10^11^ Am^−2^. At this temperature, the VW maintains its structure as it approaches the confined area [Figure 2b]. However, when the device temperature is raised to 200 K, the VW transforms into a transverse domain wall (TW), as shown in Figure 2c. The observed transformation of the vortex domain wall (VW) into a transverse domain wall (TW) at a temperature of 200 K can be directly linked to Walker breakdown. Walker breakdown occurs when the domain wall velocity exceeds a critical threshold, leading to an oscillatory motion and structural instability of the domain wall. In this study, as *M_s_* is fixed at 600 kAm^−1^ and current density remains constant at 7.5 × 10^11^ Am^−2^, the increase in temperature contributes to an overall enhancement in thermal energy, which accelerates the VW motion. At 100 K, the VW remains stable, maintaining its structure while moving through the confined region. However, at 200 K, the increased thermal activation energy leads to higher domain wall velocity, pushing the VW closer to the critical velocity associated with Walker breakdown. As a result, the VW transitions into a TW, a signature characteristic of Walker breakdown dynamics, where the domain wall structure alternates between vortex and transverse configurations due to excessive motion speed. This finding indicates that at higher temperatures, thermal fluctuations can induce an earlier onset of Walker breakdown, affecting the stability and reliability of VW-based memory systems. However, when the device temperature is set to 200 K and *M_s_* is increased to 650 kAm^−1^, the VW maintains its structure up to the constricted area, as illustrated in Figure 2d. This demonstrates that increasing *M_s_* enhances VW stability, preventing its transformation into a TW under thermal fluctuations and Walker breakdown. These observations are further validated by the m_x_ versus time plot, which compares VW behavior at two different temperatures with *M_s_* set at 600 kAm^−1^. The role of *M_s_* in reducing Walker breakdown can be understood in terms of its impact on domain wall dynamics. Higher *M_s_* strengthens dipolar interactions, increasing the energy barrier required for domain wall transformations.

At a device temperature of 100 K, the graph is smooth, showing no indications of VW transformation. In contrast, at a device temperature of 200 K, the curve shows bending at 3 ns (indicated by an arrow), corresponding to the transformation of the VW to a transverse domain wall (TW), as shown in Figure 2e. For further clarification, the VW transformation affects the DW velocity. Figure 2f shows the smooth movement of the VW at a device temperature of 100 K, while a reduction in DW velocity is observed due to VW transformation. This suggests that as the device temperature increases, the likelihood of Walker breakdown occurring also increases.

**Figure 3:**
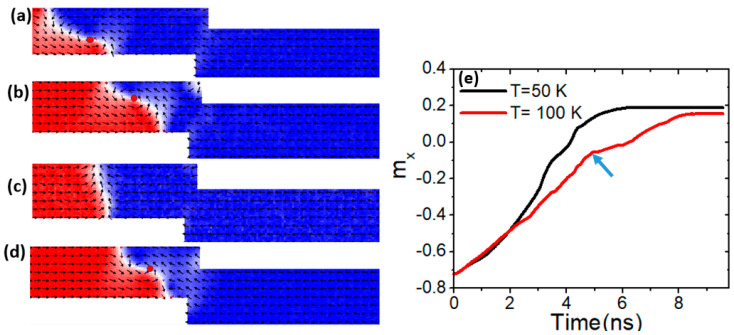
(**a**,**b**) VW dynamics in constricted nanowire (100 × 50 nm^2^) with *M_s_* = 600 kAm^−1^, a device temperature of 50 K, and a current density of 7.5 × 10^11^ Am^−2^. (**c**) VW transformation to TW under *M_s_* = 600 kAm^−1^, a device temperature of 100 K, and a current density of 7.5 × 10^11^ Am^−2^. (**d**) VW motion with *M_s_* = 650 kAm^−1^ and under a device temperature of 100 K. (**e**) m_x_ versus time for two curves with different device temperature values of 50 K and 100 K.

To further investigate the control of VW thermal transformation through adjustments in *M_s_*, simulations were conducted using confined nanowires with a constricted area of 100 × 50 nm^2^. Figure 3a illustrates the VW motion within these magnetic nanowires. Our findings indicate that with an *M_s_* of 600 kAm^−1^, the VW retains its structure as it moves under a device temperature of 50 K [Figure 3b]. Conversely, as the device temperature rises to 100 K, the VW transitions into a transverse domain wall (TW). However, by increasing *M_s_* to 650 kAm^−1^, the VW transformation is delayed, occurring at a higher temperature of 150 K.

To gain a deeper understanding, two graphs of m_x_ versus time with an *M_s_* of 600 kAm^−1^ were plotted for two different temperatures. The graph depicting VW dynamics at a temperature of 50 K (black curve) shows a smooth progression without any indication of VW transformation. However, when the device temperature is increased to 100 K, a bend appears in the graph (indicated by an arrow), indicating the onset of VW transformation [Figure 3e]. Increasing step depth (*d*) intensifies domain wall velocity, shape anisotropy gradients, and dipolar interactions, all of which contribute to Walker breakdown occurring before the VW reaches the step. This effect is particularly relevant in thermal and high-speed spintronic applications, where precise control over VW stability and motion is crucial for reliable memory operation. Therefore, as *d* increases, VW transformation occurs at lower temperatures, reducing the thermal threshold for domain wall instability.

**Figure 4:**
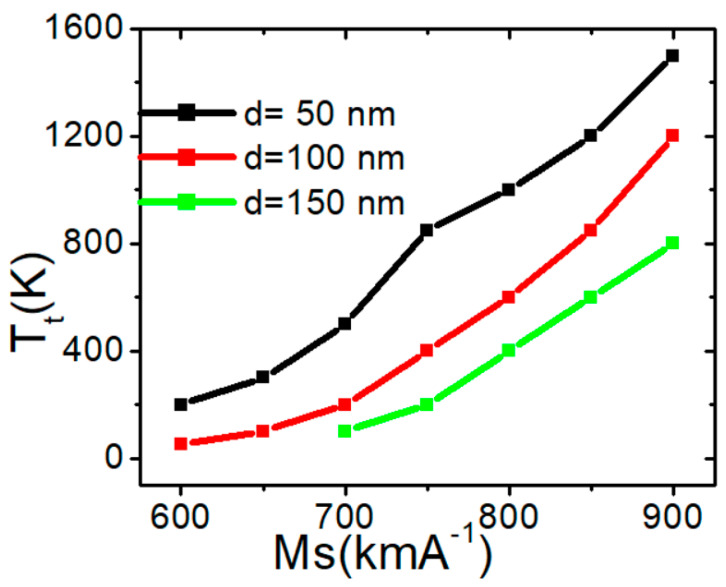
*T_t_* as a function of *M_s_* for three magnetic confined nanowires with dimensions of 50 × 50 nm^2^, 100 × 50 nm^2^, and 150 × 50 nm^2^.

A similar investigation was carried out on a nanowire with a confined region measuring 150 × 50 nm^2^, revealing that the structural stability of the VW commenced at an *M_s_* of 700 Am^−2^. At this *M_s_* value, the VW transformed into a transverse domain wall (TW) at a device temperature of approximately 45 K. Figure 4 shows the relationship between the transition temperature (*T_t_*) and *M_s_* for the three confined nanowires. The graphs indicate that the thermal structural stability of the VW is enhanced by increasing *M_s_*.

Increasing *M_s_* enhances the thermal structural stability of the VW by strengthening the stray fields surrounding it.

**Figure 5:**
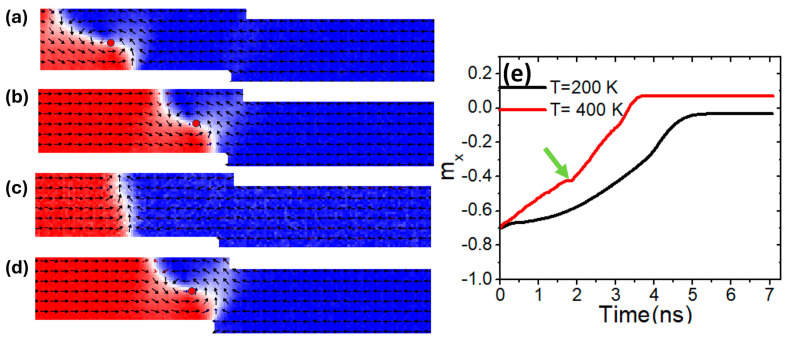
(**a**,**b**) VW dynamics in constricted nanowire (50 × 50 nm^2^) with *K_u_* = 0.5 × 10^5^ Jm^−3^, a device temperature of 200 K, and a current density of 7.5 × 10^11^ Am^−2^. (**c**) VW transformation to TW under *K_u_* = 0.5 × 10^5^ Jm^−3^, a device temperature of 400 K, and a current density of 7.5 × 10^11^ Am^−2^. (**d**) VW motion with *K_u_* = 1.5 × 10^5^ Jm^−3^ and under a device temperature of 400 K. (**e**) m_x_ versus time for two curves with different device temperature values of 200 K and 400 K.

Another magnetic property that helps maintain the thermal VW structural stability is uniaxial magnetic anisotropy (*K_u_*). The investigation was conducted using different values of *K_u_*, while *M_s_* was fixed at 800 kAm^−1^. Simulations were performed on three confined structures. First, we started with a structure of 50 × 50 nm^2^. Figure 5a illustrates the VW movement in a magnetic nanowire with *K_u_* = 0.5 × 10^5^ Jm^−3^ under a device temperature of 200 K. Under these conditions, the VW exhibited high structural stability as it moved until it reached the confined area [Figure 5b]. However, when the device temperature was raised to 400 K, the VW lost its structural stability and transformed into a transverse domain wall (TW) during its dynamics [Figure 5c]. By increasing *K_u_* to 1.5 × 10^5^ Jm^−3^, the VW regained its structural stability and successfully reached the constricted area as a VW [Figure 5d].

For a better illustration of the thermal transformation of a vortex domain wall (VW), *m_x_* versus time is plotted for two temperatures. The curve for 200 K (black curve) shows the magnetization transitioning smoothly from the positive *x*-axis direction to the negative *x*-axis. In contrast, the red curve depicts m_x_ as a function of time under a device temperature of 400 K, indicating a transformation around 2 ns, as highlighted by the green arrow in Figure 5e.

To verify that *K_u_* can improve the structural stability of a vortex domain wall (VW), a similar investigation was performed on a confined nanowire with dimensions of 100 × 50 nm^2^. A current density of 7.5 × 10^11^ Am^−2^ was applied, and the uniaxial magnetic anisotropy was maintained at *K_u_* = 0.5 × 10^5^ Jm^−3^.

**Figure 6:**
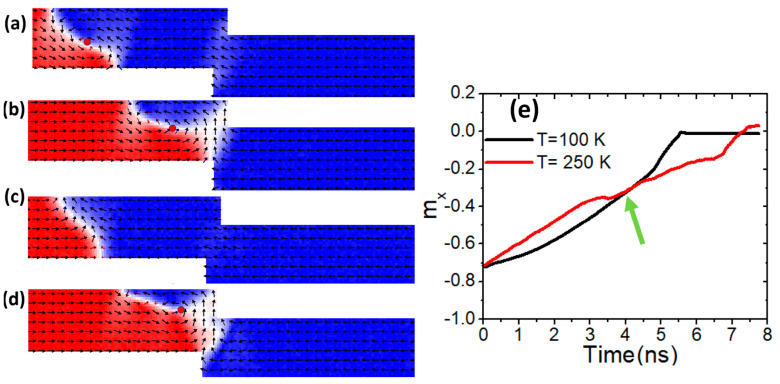
(**a**,**b**) VW dynamics in constricted nanowire (100 × 50 nm^2^) with *K_u_* = 0.5 × 10^5^ Jm^−3^, a device temperature of 100 K, and a current density of 7.5 × 10^11^ Am^−2^. (**c**) VW transformation to TW under *K_u_* = 0.5 × 10^5^ Jm^−3^, a device temperature of 400 K, and a current density of 7.5 × 10^11^ Am^−2^. (**d**) VW motion with *K_u_* = 1.5 × 10^5^ Jm^−3^ and under a device temperature of 400 K. (**e**) m_x_ versus time for two curves with different device temperature values of 200 K and 400 K.

Figure 6a illustrates the dynamics of a vortex domain wall (VW) in a magnetic nanowire with a constricted area of 100 × 50 nm^2^ at a device temperature of 100 K. In Figure 6b, the VW maintains high structural stability upon reaching the confined area at the same temperature. However, when the device temperature rises to 250 K, the VW loses its structural stability and transforms into a transverse domain wall (TW), as illustrated in Figure 6c.

By increasing *K_u_* to 1.5 × 10^5^ Jm^−3^, the VW retains its structural stability and successfully reaches the constricted area as a VW, as depicted in Figure 6d. For further clarity, the m_x_ magnetization versus time is plotted for two device temperatures. The black curve, corresponding to 100 K, shows a smooth transition during the VW’s motion. However, the red curve exhibits curling after approximately 3.5 ns, indicating the transformation of the VW into a TW, as seen in Figure 6e.

**Figure 7:**
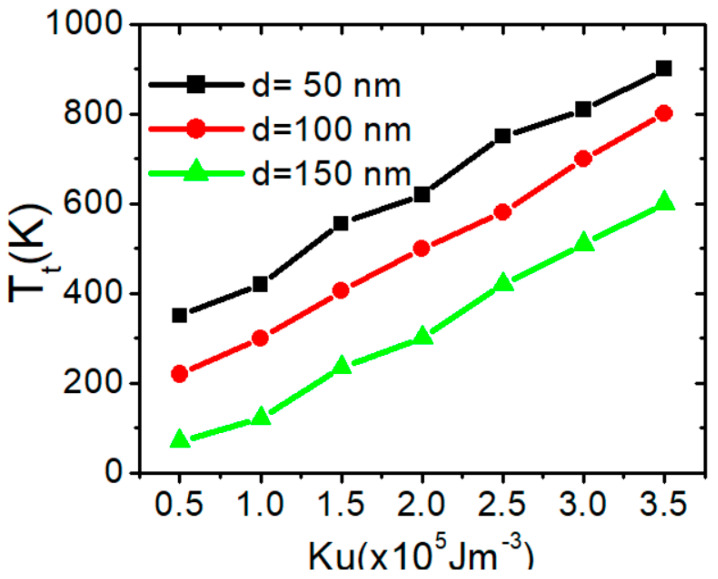
Transition temperature (T_t_) as a function of *K_u_* for three magnetic confined nanowires with dimensions of 50 × 50 nm^2^, 100 × 50 nm^2^, and 150 × 50 nm^2^.

Using the same *K_u_*, it was observed that increasing *d* makes the VW more likely to transform into a TW, and the transformation time *T_t_* decreases. Additional simulations were conducted using nanowires with a confined area of 150 × 50 nm^2^. The relationship between the transformation temperature (*T_t_*) and *K_u_* for the three confined nanowires shows a linear trend, as illustrated in Figure 7.

**Figure 8:**
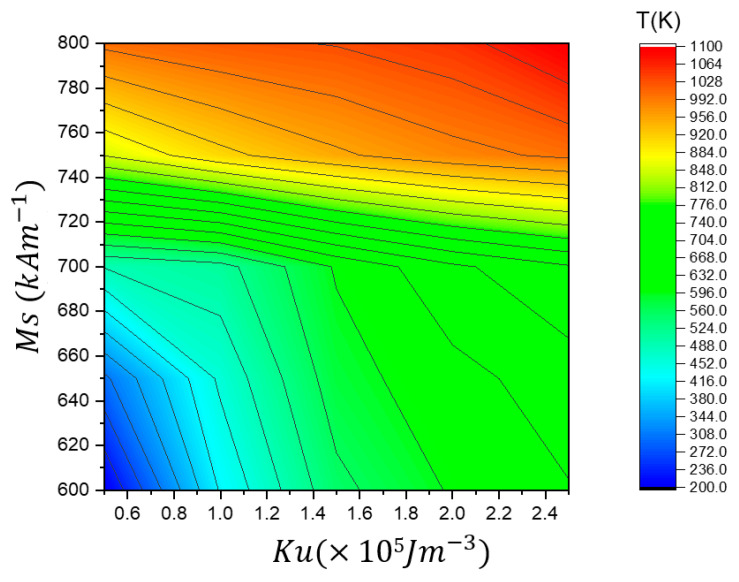
A phase diagram summarizing the effects of *M_s_* and *K_u_* on VW thermal transformation temperature (T_t_).

Figure 7 clearly shows that a higher *K_u_* value improves the thermal stability of the vortex domain wall (VW). For instance, in a nanowire with *d* of 100 nm and *K_u_* = 1.5 × 10^5^ Jm^−3^, the VW transforms into a TW at approximately 500 K. In contrast, with *K_u_* = 3 × 10^5^ Jm^−3^, the transformation occurs at 700 K. Increasing *K_u_* values raises the energy required for magnetization to deviate from its equilibrium state, thereby preventing structural deformations in the VW. However, the transformation temperature decreases as *d* increases for the same value of *K_u_*. For example, with *K_u_* = 2.5 × 10^5^ Jm^−3^, the VW transforms into a TW at 760 K in nanowires with d = 50 nm. In comparison, for nanowires with d = 100 nm and d = 150 nm, the transformation occurs at 580 K and 400 K, respectively. Figure 8 summarizes the effects of *M_s_* and *K_u_* on VW thermal transformation, providing insights into how these parameters influence VW stability for a confined nanowire with an area of 50 × 50 nm^2^.

Higher *M_s_* and *K_u_* increase the VW transformation temperature, as observed in the upper right region (red-yellow) of the phase diagram, where VW transformation occurs at higher temperatures (above 1000 K). This indicates that stronger magnetization and anisotropy enhance thermal stability, delaying the VW transition. Conversely, lower *M_s_* and *K_u_* values, represented in the lower left region (blue-green), result in VW transformation at much lower temperatures (below 400 K), suggesting that weaker magnetization and anisotropy reduce thermal stability, making the VW more susceptible to transformation at lower temperatures. The middle region (green-yellow) shows a gradual increase in transformation temperature, indicating a balanced regime where the VW maintains its structure over a wider temperature range before undergoing transformation.

While this study focuses on the micromagnetic and geometric aspects of structural and thermal stability, it is important to acknowledge that these properties are also intrinsically influenced by atomistic factors, including chemical composition, electronic interactions, and bonding characteristics. Mesoscopic and atomistic modeling approaches, such as ab initio calculations, provide valuable insights into these dependencies, complementing micromagnetic simulations in predicting domain wall behavior under thermal and mechanical stress [26,27].

### 3.2. Thermal Effects on Vortex Domain Wall (VW) Dynamics

**Figure 9:**
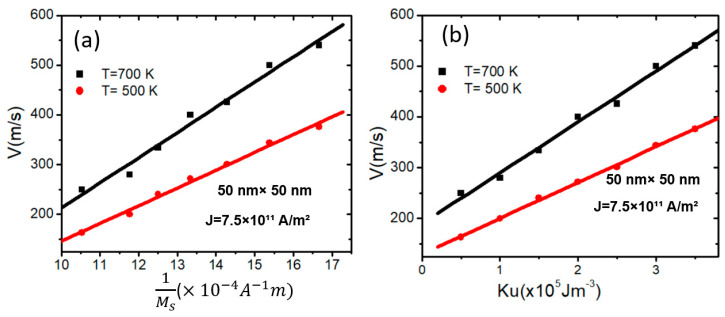
(**a**) VW velocity versus $\frac{1}{M_{s}}$ for two device temperature values and under current density value of *J* = 7.5 × 10^11^ Am^−2^ for magnetic confined nanowires with 50 × 50 nm^2^ and Ku = 3 × 10^5^ Jm^−3^. (**b**) VW velocity versus *K_u_* for two device temperature values and under current density value of *J* = 7.5 × 10^11^ Am^−2^ for magnetic confined nanowires with 50 × 50 nm^2^.

The study investigates the influence of device temperature on VW velocity at two temperature values (500 K and 700 K) for a magnetic nanowire with area dimensions of 50 × 50 nm^2^, at a fixed current density of *J* = 7.5 × 10^11^ Am^−2^. To make sure that VW is stable when varying values of *M_s_*, *K_u_* is fixed to 3 × 10^5^ Jm^−3^. Conversely, when *K_u_ is* varied, *M_s_* is fixed to 800 Am^−1^. The findings reveal that VW velocity increases linearly with increasing $M_{s}^{-1}$, as shown in Figure 9a. However, VW velocity increases linearly with *K_u_* for both temperatures, as shown in Figure 9b.

The results demonstrate the following relationships:

(5) $$v=\frac{gP\beta \mu_{b}K_{u}}{2e\alpha M_{s}}J$$

where g is the Lande factor, *P* represents spin polarization, β is the non-adiabatic parameter, $\mu_{b}$ denotes the Bohr magnetron, J signifies the current density, e corresponds to the carrier charge, α is the Gilbert damping factor, and *M_s_* represents the saturation magnetization [36].

Additionally, it was found that the VW has a correlation with device temperature, whereas the VW increases by increasing the device temperature. The relationship between VW velocity and device temperature is often described by a thermally activated process, where the VW velocity depends on temperature *T* according to the following:

(6) $$v=v_{0}exp(-\frac{E_{b}}{K_{B}T})$$

where $E_{b}$ is the energy barrier, $K_{B}$ is the Boltzmann constant, and T is the device temperature [37].

### 3.3. Thermal Control of VW Pinning and Depinning

Additional investigations were carried out to analyze the thermal pinning and depinning of the vortex domain wall (VW) as it approached the confined region, with variations in magnetic properties. The thermal pinning behavior was studied using nanowires measuring 50 × 50 nm^2^.

**Figure 10:**
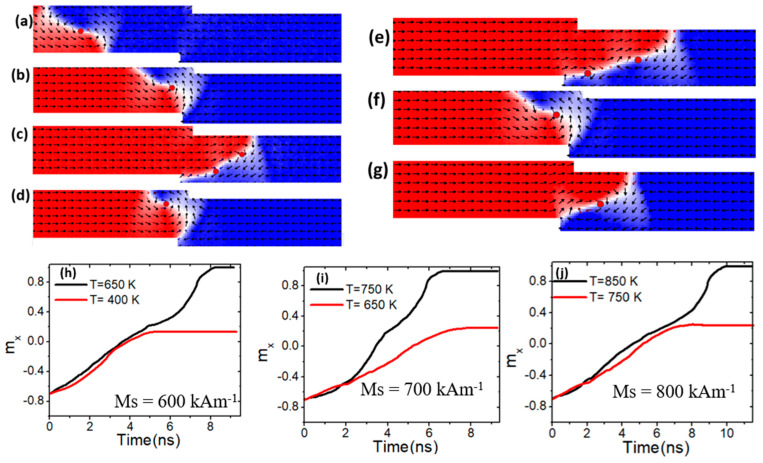
(**a**) Dynamics of vortex domain walls (VW) dynamics in confined magnetic nanowires with 50 × 50 nm^2^, under a current density value of 7.5 × 10^11^ Am^−2^ and a device temperature of 400 K. (**b**,**c**,**h**) depict VW pinning and depinning in confined magnetic nanowires with 50 × 50 nm^2^, *M_s_* of 600 kAm^−1^, and device temperature of 400 K and 650 K. (**d**,**e**,**i**) VW pinning and depinning in confined magnetic nanowires with 50 × 50 nm^2^, *M_s_* of 700 kAm^−1^, and device temperature of 650 K and 750 K. (**f**,**g,j**) show VW pinning and depinning in confined magnetic nanowires with 50 × 50 nm^2^, *M_s_* of 800 kAm^−1^, and device temperature of 750 K and 850 K.

At first, the VW was propelled toward the confined area by applying a current density, as illustrated in Figure 10a, where it became pinned [Figure 10b]. The device temperature was then gradually raised to determine the conditions under which the VW depinned and migrated to the edge of the nanowire. The depinning process was evaluated across different temperatures and *M_s_* values. At a current density of 7.5 × 10^11^ Am^−2^, a device temperature of 400 K, and *M_s_* value of 600 kAm^−1^, the VW remained pinned within confined region, as illustrated in Figure 10b. The red curve in Figure 10h depicts the normalized magnetization (*m_x_*) versus time. However, at temperatures of 650 K and above, thermal pinning weakened, resulting in depinning, as shown in Figure 10h (black graph). As a result of this device’s depinning temperature and low *M_s_* value, VW transforms into an antivortex wall [Figure 10c]. As a result of the combined effects of thermal fluctuations, low *M_s_*, weakened exchange interactions, and reduced domain wall pinning potential at the step [Figure 10c], higher temperatures facilitate VW to AVW transformations by lowering the energy barrier for nucleation. Additionally, low *M_s_* reduces vortex stability, making the AVW configuration more favorable.

In contrast, by increasing *M_s_* to 700 kAm^−1^, it is found that VW was pinned when it reached the confined area under a device temperature of 650 K, as illustrated in Figure 10d,i (red graph). With this value of *M_s_*, the VW depinning process happened when the temperature reached 750 K and above, as illustrated in Figure 10e,i (black graph). As the *M_s_* was increased to 800 kAm^−1^, the VW stayed pinned in the confined area. Under this value of *M_s_*, the VW started to depinning the constricted area at operating temperatures of 850 K and above, as shown in Figure 10g.

**Figure 11:**
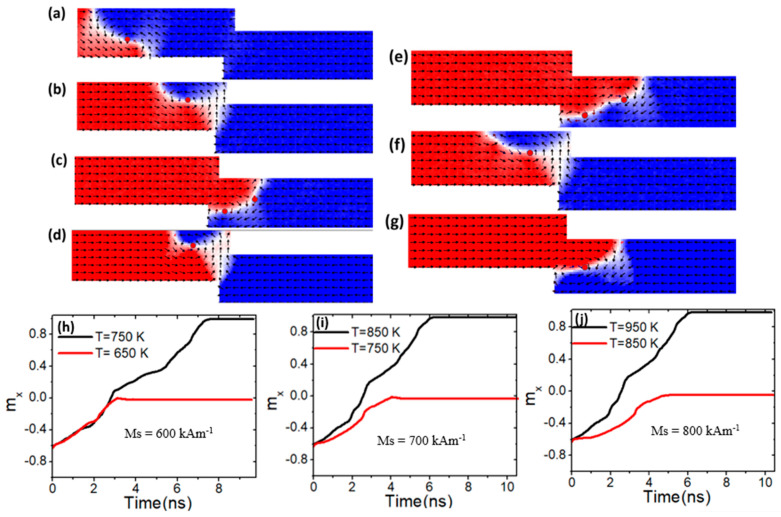
(**a**) Dynamics of vortex domain walls (VW) dynamics in confined magnetic nanowires with 100 × 50 nm^2^, under a current density value of 7.5 × 10^11^ Am^−2^, and a device temperature of 650 K. (**b**,**c**,**h**) illustrate VW pinning and depinning in confined magnetic nanowires with 100 × 50 nm^2^, *M_s_* of 600 kAm^−1^, and device temperature of 650 K and 750 K. (**d**,**e**,**i**) VW pinning and depinning in confined magnetic nanowires with 100 × 50 nm^2^, *M_s_* of 700 kAm^−1^, and device temperature of 750 K and 850 K. (**f**,**g**,**j**) show VW pinning and depinning in confined magnetic nanowires with 100 × 50 nm^2^, *M_s_* of 800 kAm^−1^, and device temperature of 850 K and 950 K.

To gain a deeper understanding of varying *M_s_* values on VW thermal pinning, a nanowire with confined area dimensions of 100 × 50 nm^2^ was used. The VW dynamics as it approached the constricted area are depicted in Figure 11a, while Figure 11b illustrates VW pinning within the pinning area. With an *M_s_* value of 600 kAm^−1^, it was discovered that VW exhibits high thermal pinning at temperatures below 750 K, as shown in Figure 11b and the black graph for 650 K in Figure 11h. However, at a device temperature of 750 K and above, VW depinned the confined area with two vertices to the end of the nanowire, as shown in Figure 11c and the red graph in Figure 11h. When the *M_s_* value was raised to 700 kAm^−1^, VW exhibited more thermal pinning under this device temperature (750 K), as depicted in Figure 11d and the red graph in Figure 11i. In contrast, VW started to depin the confined area by increasing the device temperature to 850 K and above, as shown in Figure 11c and black plotting in Figure 11i. VW gained its thermal stability in this area by increasing the *M_s_* value to 800 KAm^−1^, as shown in Figure 11f and the red graph in Figure 11 (j). However, with this value of *M_s_*, VW lost its pinning stability by increasing device temperature to 950 K and above [Figure 11g and black plotting in Figure 11j]. It is noted that VW becomes more stable in its structure by depinning with one vortex core compared to the *M_s_* value of 600 kAm^−1^ and 700 kAm^−1^ that VW depinned with two vortex cores.

**Figure 12:**
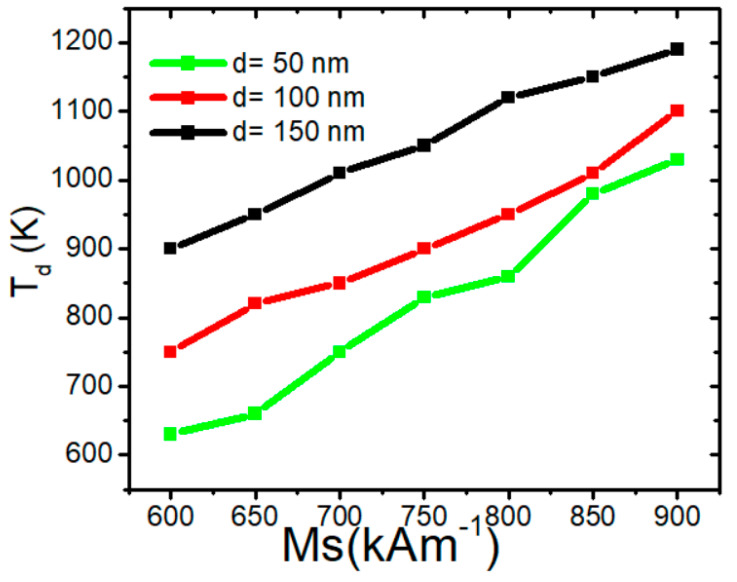
VW depinning temperature in confined nanowires with varying of *M_s_* with area dimensions of 50 × 50 nm^2^, 100 × 50 nm^2^, and 150 × 50 nm^2^.

The graph in Figure 12 represents the dependence of the depinning temperature (*T_d_*) on the saturation magnetization (*M_s_*) for three different nanowire values of d (50 nm, 100 nm, and 150 nm). Increasing *d* raises the depinning temperature due to stronger magnetic interactions, including enhanced dipolar coupling, increased shape anisotropy, and higher exchange energy constraints, which collectively stabilize VW and increase the energy required for depinning. The linear trends and their variations across d provide insight into the thermal stability and magnetic properties of the nanowires. This gives evidence that *M_s_* plays a critical role in determining its resistance to temperature-induced depinning. Increasing *M_s_* leads to a rise in VW thermal structural stability due to dipolar interactions, which results in Stronger localized pinning sites due to amplified magnetostatic energy. Higher stray field gradients create an increased energy barrier for VW depinning. Enhanced domain wall rigidity makes the VW more stable against external perturbations.

**Figure 13:**
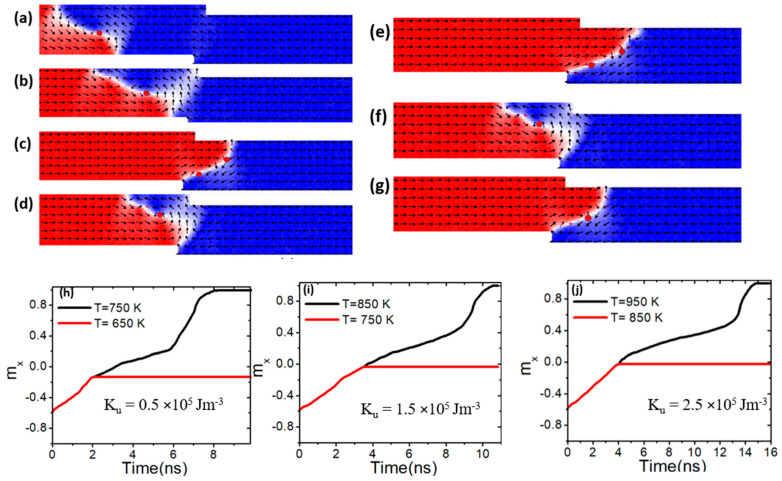
(**a**) Dynamics of vortex domain walls (VWs) in confined magnetic nanowires with 50 × 50 nm^2^, under a current density value of 7.5 × 10^11^ Am^−2^ and a device temperature of 400 K. (**b**,**c**,**h**) illustrate VW pinning and depinning in confined magnetic nanowires with 50 × 50 nm^2^, *K_u_* of 0.5 × 10^5^ Jm^−3^, and device temperature of 650 K and 750 K. (**d**,**e**,**i**) depict VW pinning and depinning in confined magnetic nanowires with 50 × 50 nm^2^, 1.5 × 10^5^ Jm^−3^, and device temperature of 750 K and 850 K. (**f**,**g**,**j**) show VW pinning and depinning in confined magnetic nanowires with 50 × 50 nm^2^, *K_u_* of 2.5 × 10^5^ Jm^−3^, and device temperature of 850 K and 950 K.

*K_u_* is another property influencing the thermal pinning and depinning of vortex domain walls (VWs) in confined magnetic nanowires. In this analysis, the step length was adjusted while keeping *M_s_* fixed at 800 kAm^−1^. Figure 13a depicts VW dynamics in a confined nanowire with dimensions of 50 nm × 50 nm. Figure 13b and the red graph in Figure 13h demonstrate VW pinning at a *K_u_* value of 0.5 × 10^5^ Jm^−3^ and a device temperature of 650 K. It was observed that VW began to depin from this step size and *K_u_* value starting at 750 K, as shown in Figure 13c and the black graph in Figure 13h. When the *K_u_* value was increased to 1.5 × 10^5^ Jm^−3^, thermal VW depinning occurred at 850 K [Figure 13e], and at 950 K when *K_u_* was further increased to 2.5 × 10^5^ Jm^−3^ [Figure 13g]. These findings suggest that increasing *K_u_* enhances VW thermal stability, reducing depinning and enabling the VW to remain pinned in the confined region at higher temperatures.

**Figure 14:**
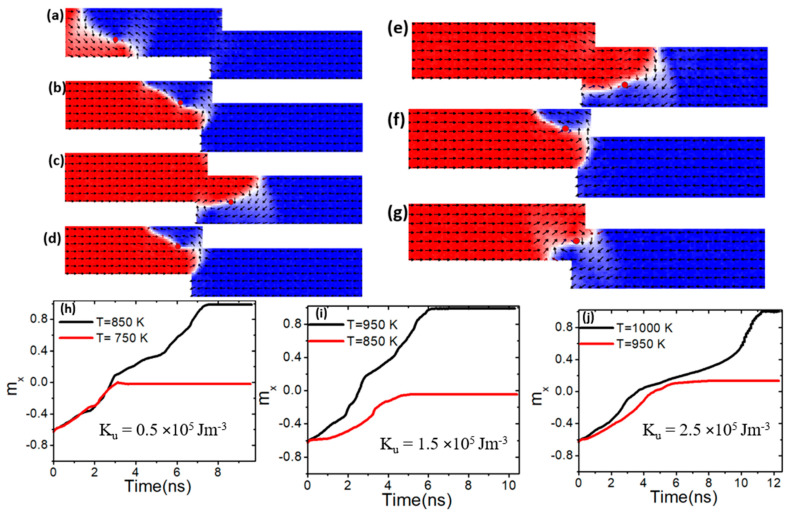
(**a**) VW dynamics in confined magnetic nanowires with 100 × 50 nm^2^, under a current density value of 7.5 × 10^11^ Am^−2^ and a device temperature of 750 K. (**b**,**c**,**h**) VW pinning and depinning in confined magnetic nanowires with 100 × 50 nm^2^, *K_u_* of 0.5 × 10^5^ Jm^−3^, and a device temperature of 750 K and 850 K. (**d**,**e**,**i**) VW pinning and depinning in confined magnetic nanowires with 100 × 50 nm^2^, 1.5 × 10^5^ Jm^−3^, and a device temperature of 850 K and 950 K. (**f**,**g**,**j**) VW pinning and depinning in confined magnetic nanowires with 100 × 50 nm^2^, *K_u_* of 2.5 × 10^5^ Jm^−3^, and a device temperature of 950 K and 1000 K.

To enhance thermal pinning in the confined region, a nanowire with pinning area dimensions of 100 × 50 nm^2^ was employed while maintaining the same *K_u_* values. The results indicate that the VW exhibited increased resistance to depinning within the confined area as the device temperature rose. At a *K_u_* value of 0.5 × 10^5^ Jm^−3^, the VW started to depin from the confined region at a device temperature of 850 K, as shown in Figure 14c and the black curve in Figure 14h. For a *K_u_* value of 1.5 × 10^5^ Jm^−3^, the VW remained pinned in the confined region at 850 K but started to move through the constricted region at a higher temperature of 950 K, as depicted in Figure 14e and the black plot in Figure 14i.

When *K_u_* was increased to 2.5 × 10^5^ Jm^−3^, thermal depinning of the VW occurred at an even higher temperature of 1000 K, as shown in Figure 14g and the black graph in Figure 14j. These findings suggest that VW thermal pinning strengthens as *K_u_* increases, enabling the VW to remain pinned longer and depin only at higher temperatures.

**Figure 15:**
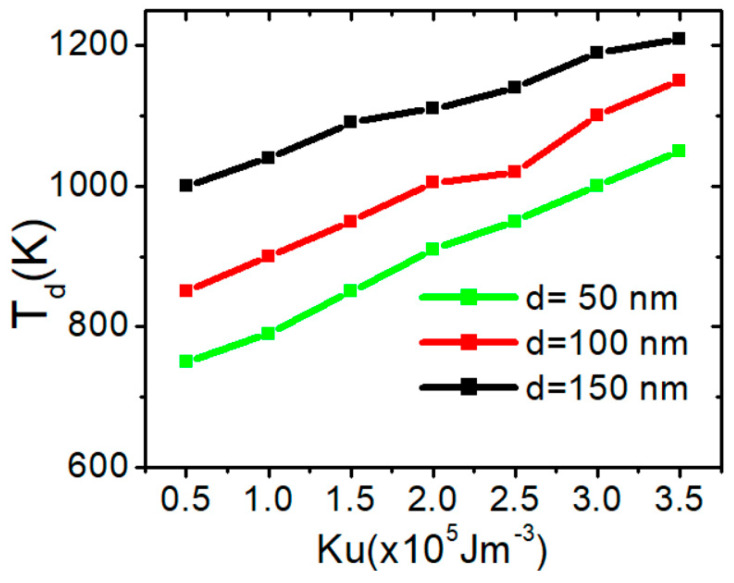
VW depinning temperature in confined nanowires with varying Ku with area dimensions of 50 × 50 nm^2^, 100 × 50 nm^2^, and 150 × 50 nm^2^.

Here is a summary of the influence of *K_u_* and *d* on the thermal stability of vortex domain walls (VWs). Figure 15 illustrates the relationship between depinning temperature (*T_d_*) and *K_u_* for three nanowires with varying *d* values under two different current density. The results indicate that *T_d_* has a linear relationship with *K_u_*, increasing as *K_u_* increases. Increasing *K_u_* results in a higher energy barrier for VW depinning, creating a more rigid domain wall that maintains its structure even under external forces or temperature variations. This also enhances resistance to thermal fluctuations, preventing unwanted domain wall transformations.

**Figure 16:**
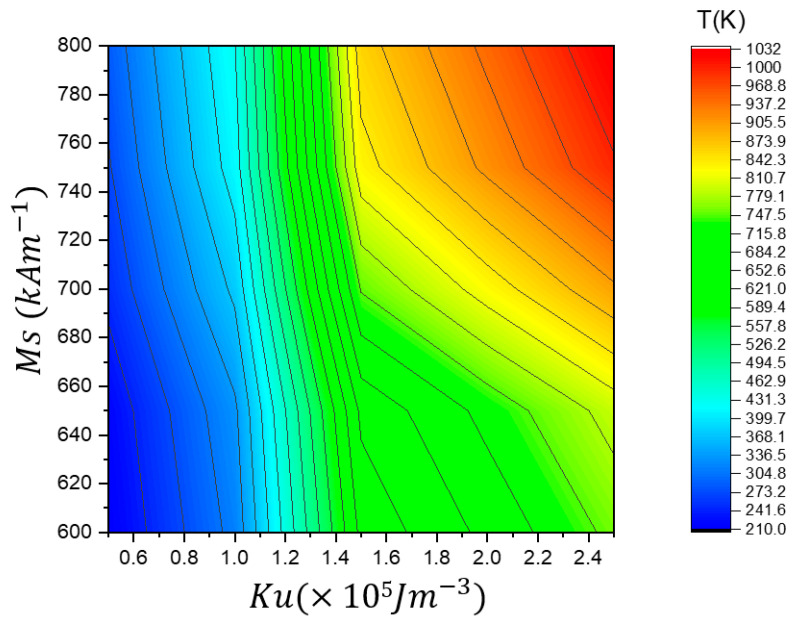
A phase diagram illustrating the VW depinning temperature (*T_d_*) as a function of *M_s_* and *K_u_* in the stepped region of 50 × 50 nm^2^.

In Figure 16, the phase diagram summarizes the vortex domain wall (VW) depinning temperature (*T*_d_) in the stepped region as a function of *M_s_* and *K_u_*. The color gradient represents the depinning temperature (*T*_d_), providing insights into how different magnetic properties influence the thermal stability of VW motion through the stepped area of 50 × 50 nm^2^.

This phase diagram summarizes the vortex domain wall (VW) depinning temperature (*T_d_*) in the stepped region as a function of *M_s_* and *K*_u_. The color gradient represents the depinning temperature (*T_d_*), providing insights into how different magnetic properties influence the thermal stability of VW motion through the stepped area. Higher *M_s_* and *K*_u_ increase the VW depinning temperature, as seen in the upper right region (red/yellow), where depinning occurs at temperatures above 1000 K. This indicates that stronger magnetization and anisotropy enhance pinning strength, requiring higher thermal energy to depin the VW. In contrast, lower *M_s_* and *K*_u_ lead to earlier depinning, as represented in the lower left region (blue/green), where VW depinning occurs at much lower temperatures (below 400 K), suggesting that weak magnetization and low anisotropy reduce the pinning energy, making the VW more likely to depin at lower thermal energies. The middle region (green-yellow) represents a gradual increase in depinning temperature, indicating a balance where VW remains pinned over a broader temperature range before depinning. Optimizing *M_s_* and *K*_u_ is crucial for designing memory devices with controlled VW motion, as low values of *M_s_* and *K*_u_ can lead to premature depinning, reducing the stability of stored information, while higher values enhance pinning strength, reducing the likelihood of thermal fluctuations causing unintended VW depinning.

As nanowire devices are scaled up for higher-density memory storage, the interplay between pinning energy, thermal stability, and device geometry becomes increasingly important. The results of this study suggest that maintaining high saturation magnetization and uniaxial magnetic anisotropy at larger scales will be essential to preserve the thermal stability of VWs, which is necessary for minimizing data loss and improving the performance of high-density storage devices

From a fabrication perspective, the findings from this study could guide the development of new materials or geometries for nanowire-based memory devices. For instance, by tailoring the uniaxial magnetic anisotropy and saturation magnetization during the fabrication of nanowires, manufacturers can control the pinning strength of domain walls. This would allow for the production of devices that are not only thermally stable but also more energy-efficient and reliable in harsh operating environments.

## 4. Conclusions

This study has demonstrated that vortex domain wall (VW) thermal stability in constricted magnetic nanowires is significantly influenced by saturation magnetization (*M_s_*) and uniaxial anisotropy (*K*_u_). The results highlight that higher values of *M_s_* and *K*_u_ enhance resistance to thermal transformations, allowing VWs to remain stable at device temperatures exceeding 500 K, which is essential for improving the performance and reliability of nanoscale magnetic memory devices.

Temperature also plays a crucial role in VW dynamics. The velocity of VWs increases with decreasing *M_s_* and increasing *K*_u_, following a nearly linear trend. At higher temperatures, such as 700 K, the velocity is consistently greater than at 500 K, demonstrating that thermal energy accelerates domain wall motion. The effect of *K*_u_ on VW velocity is particularly strong, as higher anisotropy reduces internal magnetic disorder, leading to more controlled and predictable VW motion. In contrast, lower *M_s_* weakens dipolar interactions, allowing for smoother and faster VW propagation under a constant current density.

In constricted nanowire regions, increasing *M_s_* or *K*_u_ raises the depinning temperature, exceeding 800 K in some cases. This stabilization effect suggests that precisely tuning magnetic properties can significantly enhance VW-based memory system reliability. Additionally, the stepped nanowire geometry, particularly the step depth (*d*), plays a critical role in VW stability. Our findings show that at 50 nm, the VW remains pinned up to 800 K for certain values of *M_s_* or *K*_u_. Increasing d beyond 100 nm further delays depinning, exceeding 900 K, underscoring the importance of geometric constraints in optimizing VW behavior.

These findings demonstrate that carefully optimizing magnetic parameters and nanowire geometries can lead to more robust, energy-efficient spintronic memory devices capable of withstanding thermal fluctuations. Overall, this study provides valuable insights into the design and development of next-generation nanowire-based storage technologies, improving thermal stability, data retention, and energy efficiency.

## Data Availability

Data is contained within the article or Supplementary Materials.

## Supplementary Materials

The following supporting information can be downloaded at: https://www.mdpi.com/article/10.3390/nano15050372/s1. Refs. [38,39,40,41,42,43] are cited in the Supplementary Materials.
