# Supplementary material for: Harnessing Magnetic Properties for Precision Thermal Control of Vortex Domain Walls in Constricted Nanowires
Source: Nanomaterials (Basel). 2025 Feb 27;15(5):372. doi: 10.3390/nano15050372 (PMC11901696; doi:10.3390/nano15050372)
Supplement: Supplementary file 1 [file nanomaterials-15-00372-s001.zip › nanomaterials-3501053-supplementary.pdf]

Supplementary (Harnessing Magnetic Properties for Precision Thermal Control of Vortex Domain Walls in Constricted Nanowires)

Mohammed Al Bahri*, Salim Al-Kamiyani

Department of Basic and Applied Sciences, A'Sharqiyah University, P.O. Box 42, PC 400, Ibra, Oman.

*Corresponding Author Email: mohammed.albahri@asu.edu.om

After reviewing several publications on controlling DW dynamics and pinning in magnetic nanowires [1][2][3][4][5][6], the authors identified a research gap in the parameters that affect vortex domain wall (VW) transformation and pinning sites. The authors argue that the VW structure, temperature, and stability at the pinning site are three key factors that significantly influence the performance of magnetic nanodevices.

To investigate this, the authors used the Object-Oriented MicroMagnetic Framework (OOMMF) to test their hypothesis. The OOMMF software operates based on the Landau-Lifshitz-Gilbert (LLG) equation with spin-transfer torque (STT) terms and a thermal field. A C++ file was used with various commands related to device dimensions, magnetic properties, and a specific command for setting the device temperature.

In this simulation, a stepped nanowire composed of in-plane magnetic materials was employed to analyze the impact of these factors on domain wall dynamics and pinning behavior.

Findings supports

1- The type of constricted nanowire

Conventionally, triangular notches are used to study pinning areas[6]. However, in this study, we used a stepped scheme. The stepped nanowire offers several advantages, such as:

- The pinning strength can be adjusted by the depth of the conventional notch. In contrast, with the stepped area, it can be adjusted either by the stepped area depth (d) or the stepped area length (λ), as shown in Figure S1.

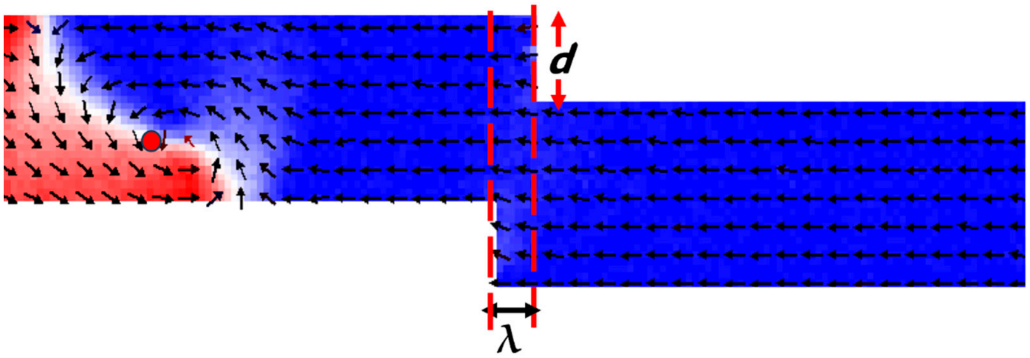

Figure S1. Stepped nanowire. Vortex domain wall VDW is nucleated at a critical current density and propagates towards the stepped region

- The conventional triangular notches include sharp edges at the middle, which contribute to VW transformation. In contrast, the stepped scheme does not include sharp edges, allowing the VW to move smoothly through the constricted area during the pinning and depinning process without transformation. This results in VW depinning with high structural stability, as shown in Figure S2.

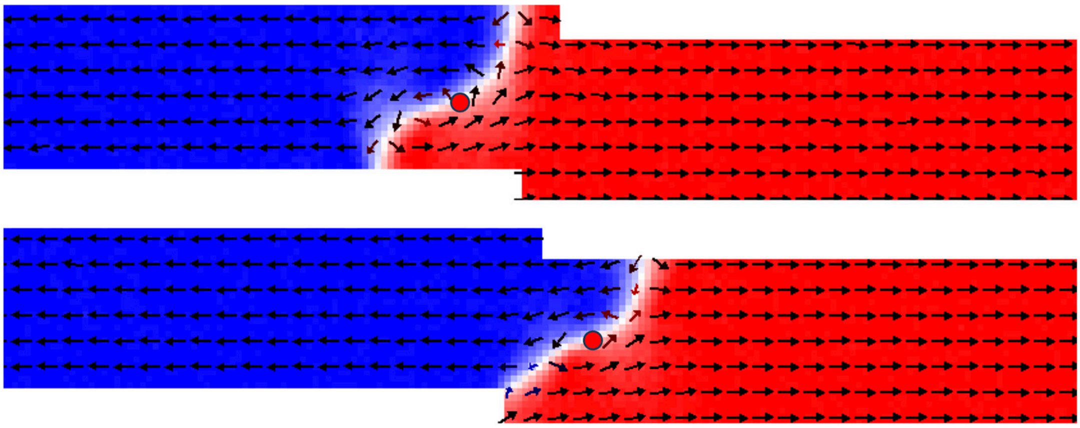

Figure S2. Vortex domain wall (VW) dynamics and structural stability in magnetic stepped nanowire

- The stepped or constricted regions, allowing for multi-bit storage, which significantly enhances memory capacity as shown in Figure S3. However, conventional notches restrict DW trapping to a single, localized position.

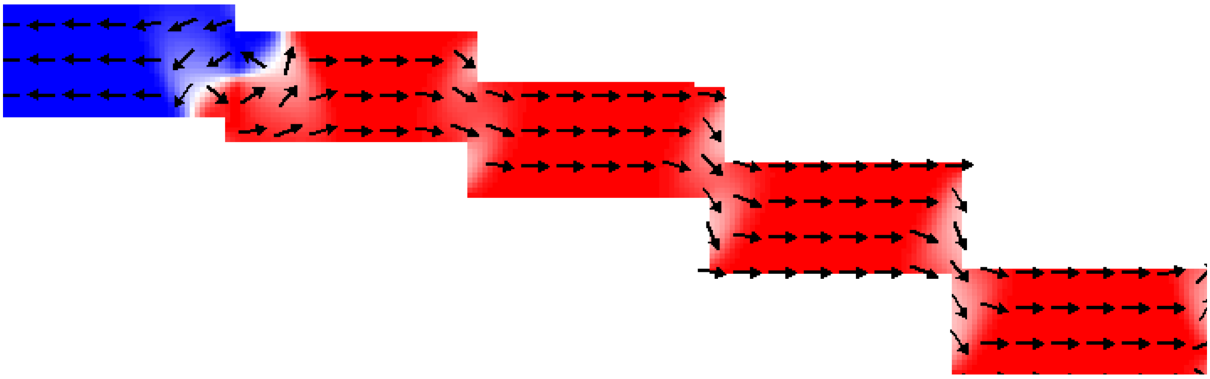

Figure S3: Multi-bit stepped nanowire

2- Impact of increasing temperature on VW structural stability

This study focuses on the effects of temperature on VW transformation and how it increases Walker breakdown. In contrast, previous studies primarily examined the effects of increasing

the magnetic field or current density, and these studies were conducted at 0 K. However, our simulations are carried out at different temperatures.

Our results reveal the extent to which temperature influences VW transformation, with these effects becoming significant at room temperature or higher, making it relevant for practical applications. We found that VW transformation occurs beyond 500 K for certain values. We found that, for certain parameter values of Ms or Ku, VW transformation occurs beyond 500 K. For example, with Ms=800kAm^{-1}, transformation with 600 K. Here the source data which used to plot T_t as a function of Ms as shown in Figure 4 in the manuscript.

Table S1: T_t as a function of M_s for three magnetic confined nanowires with dimensions of 50 × 50 nm^2, 100 × 50 nm^2, and 150 × 50 nm^2

Ms	d=50 nm	d=100 nm	d=150 nm
600	200	50	--
650	300	100	--
700	500	200	100
750	850	400	200
800	1000	600	400
850	1200	850	600
900	1500	1200	800

3- Impact of increasing temperature on VW pinning stability

The previous studies focused on investigating VW pinning stability by increasing the magnetic field or current density at 0 K[5][6]. However, this study focuses on VW depinning under the effects of temperature with a fixed current density. Our results show that VW depinning occurs at temperatures higher than room temperature, making it applicable to storage applications. For example, with Ms=600 kA/m, the VW depinning temperature is around 600 K.

4- Impact of developing magnetic materials on VW thermal structural stability and pinning

Previous studies focused on enhancing VW structural stability and pinning by modifying nanowire dimensions, such as width, thickness, and constriction geometry. While geometry can influence VW behaviour, it has limitations, particularly in controlling temperature-dependent effects and ensuring long-term stability under thermal fluctuations.

In contrast, our study takes a material-based approach by investigating the effects of magnetic properties, such as saturation magnetization (Ms) and magnetic anisotropy (Ku) in improving VW structural stability and pinning behaviour against device temperature. We found that increasing Ms or Ku enhances VW thermal structural stability and pinning behavior.

Here is the source used for plotting Figure 12 in the manuscript as an example.

Table S2: Vortex domain wall (VW) depinning temperature (T_d) as a function of M_s in the stepped region of 50 × 50 nm^2, 100 × 50 nm^2, and 150 × 50 nm^2.

Ms	d=50 nm	d=100 nm	d=150 nm
600	630	750	900
650	660	820	950
700	750	850	1010
750	830	900	1050
800	860	950	1120
850	980	1010	1150
900	1030	1100	1190

Here is the data used to plot the phase diagram (Figure 8 in the manuscript). The phase diagram illustrates the effects of improved magnetic properties (Ms and Ku) on thermal VW stability by showing the device temperature at which transformation occurs.

Table S3. The effects of M_s and K_u on VW thermal transformation temperature (T_t).

Ms	ku=0.5	ku=1	ku=1.5	ku=2	ku=2.5
600	200	420	550	600	630
650	300	460	580	620	650
700	490	510	600	660	700
750	850	910	950	980	1000
800	1000	1020	1030	1050	1100

Here is the source data for plotting the phase diagram in Figure 16 of the manuscript. This phase diagram summarizes the VW depinning temperature with the enhancement of Ms and Ku .

Table S4: Vortex domain wall (VW) depinning temperature (T_d) as a function of M_s and K_u in the stepped region of 50 × 50 nm^2.

Ms	ku=0.5	ku=1	ku=1.5	ku=2	ku=2.5
600	210	315	630	693	756
650	220	330	660	726	792
700	250	375	750	825	900
750	270	415	830	913	996
800	285	430	860	946	1032

Reference List:

[1] M. Hayashi, and L. Thomas et al., " Dependence of Current and Field Driven Depinning of Domain Walls on Their Structure and Chirality in Permalloy Nanowires," PRL, vol. 97, pp. 207205, 2006.

[2] D.S. Eastwood, L.K. Bogart and D. Atkinson, " Scaling Behaviour of Chirality Dependent Domain Wall Pinning in Planar Nanowires," ACTA PHYSICA POLONICA A, vol. 118, pp. 719-722, 2010.

[3] A. Fert et al., "Spin torque and magnetic domain walls in nanowires," Journal of Magnetism and Magnetic Materials, vol. 295, pp. 111-118, 2005.

[4] M. Boulle et al., "Spin torque and thermal effects in magnetic nanowires," Nature Physics, vol. 4, pp. 473-477, 2008.

[5] WU Yong, XU XiaoGuang et al., " Current-induced domain wall motion in magnetic nanowires with different dimensions," Physics, Mechanics & Astronomy, vol. 55, pp. 2030–2032, 2012.

[6] H. Y. Yuan and X. R. Wang, " Domain wall pinning in notched nanowire", Phys. Rev. B, vol. 89, 054423, 2014.
